# The Comparative Germicidal Action of Some Disinfectants

**Published:** 1901-02

**Authors:** 


### THE COMPARATIVE GERMICIDAL ACTION OF SOME DISINFECTANTS.

The *Lancet* of June 23, 1900, has in it a paper by Burgess detailing some experiments made by him to determine the relative activity of various antiseptics. The specific germ selected for these experiments was the bacillus coli communis, a bacillus which occurs normally in the human intestinal tract, and in health appears to be innocuous, but which under abnormal conditions may become markedly pathogenic. This bacillus possesses some very distinct advantages for the purpose of this investigation; it is extremely easy to obtain it in large quantity, owing to the profuse nature of its growth on potato; it has many characteristic reactions, by which it can be readily distinguished from other bacilli, and by applying these at different stages in the course of the experiments it is possible to establish the purity of the cultures throughout; while, since it has, for a non-sporing bacillus, a comparatively high power of resistance to germicides, any results obtained with it may be held to apply even more forcibly in the case of other non-sporing bacilli, at any rate such as are pathogenic.

The bacillus was isolated from the stools of a young girl suffering from enteric fever by the method of successive gelatin plates. A pure culture having been obtained, and proved by means of the characteristic reactions to the bacillus coli communis, a stock culture was made on the sloped surface of agar-peptone bouillon in tubes, and from these subcultures on potato were taken as required. These were made on the surface of freshly prepared sterilized potato medium, and were incubated at 37° C. for ten days, when a profuse dark-brown growth, very characteristic of this bacillus, was found to have formed. This was removed with a thick sterilized platinum wire (care being taken not to carry along with it any of the potato medium), and mixed in a sterilized glass capsule with a little sterilized peptone bouillon so as to form a thick emulsion, into which the silk threads were immersed. These threads were cut from the finest "Turner's plaited silk," each being exactly one inch long, and were sterilized in a Koch's apparatus for one and a half hours in Petri dishes, in which they were afterwards dried and kept till required for use. After immersion in the emulsion for ten minutes the threads were transferred with sterilized forceps to a Petri capsule, in which they were partially dried in the dark at a temperature of 24° C. for three hours by placing them in the "cool" incubator. They were next transferred to the disinfectant solutions to be tested, and after remaining in these for the allotted time were lifted out with a sterilized platinum hook, washed well in sterilized distilled water in order to remove any trace of disinfectant adhering to them, and placed each in a separate tube of sterilized peptone bouillon. These tubes were incubated at 37° C. for ten days, being examined each twelve hours for the first forty-eight hours, and after that daily. If no growth occurs, the bouillon is perfectly clear at the end of this time; if it does occur the bouillon begins to show signs of turbidity at a period varying from twelve hours, or even less, to seventy-two hours. The presence or absence of growth is further shown by microscopical examination, and by the inoculation of gelatin plates. If no growth had taken place at the end of ten days, it was concluded that all the bacilli present on the thread had been killed by the disinfectant solution. In every case a control experiment was made precisely as above, except that sterilized distilled water replaced the disinfectant solution, and in each instance growth was found to be present at the end of twelve hours.

Objection may possibly be raised to the above method on the ground that some of the disinfectant solution might be carried over on the thread into the bouillon, and so, by its antiseptic or inhibitory action, prevent the development of bacilli in the thread which were not actually killed by it. To guard against this, the threads were, as already stated, well washed in sterilized distilled water after removal from the action of the disinfectant, and as a further precaution all those tubes which exhibited no growth at the end of ten days were reinoculated from an emulsion of bacilli prepared as above and again placed in the incubator. In every such case growth occurred at the end of twelve hours, thus proving that the bouillon in these tubes was still a suitable medium for the development of the organism, and that their previous sterility was due, not to any transferred disinfectant, but to the fact that all the bacilli on the thread had been completely destroyed.

In order to establish a fair comparison between the germicidal effects of the various disinfectants tested, Dr. Burgess says he decided to allow the threads to remain in solutions of them for certain definite lengths of time, and to ascertain the weakest strength of any given disinfectant which would destroy all the bacilli on the threads in these times respectively,—viz., one minute, five minutes, ten minutes, thirty minutes, and one hour. For each strength of a disinfectant solution acting for a given time (*e.g.*, carbolic acid 1 to 20, acting for five minutes) six threads were used, each being subsequently placed in a separate tube of bouillon and incubated. In by far the majority of cases the result was the same in each of the six tubes, either positive or negative, showing that every care had been taken to prevent contamination of the threads in the process of transferrence from the disinfectant solution to the bouillon tubes. But occasionally, when approaching the limit of the germicidal power of the particular strength of disinfectant used, there was some discrepancy,—*e.g.*, five tubes might give a positive and one a negative reaction, or *vice versa*, in which case the verdict of the majority was taken to be the correct one. If, however, the discrepancy was more than this,—*e.g.*, four and two, or three and three,—the experiment was repeated with a second set of six threads, and the majority was taken.

The following substances were tested: biniodide of mercury, perchloride of mercury, chlorinated lime, formaldehyde, lysol, carbolic acid, izal, medical izal, creolin, Jeyes's sanitary fluid, Walker's I. X. L. disinfectant fluid, Condy's fluid, "sanitas" fluid, and boric acid. These experiments involved the inoculation and culture of some two thousand tubes of bouillon, and were conducted in the laboratory of the Manchester Union Hospital, Crumpsall, during six months of Burgess's tenure of the resident medical officership there.

In interpreting these results Burgess wishes it to be clearly understood that they are merely relative, and can only be held to be absolute under the exact conditions laid down for these experiments,—*i.e.*, where the bacilli are freely exposed to the action of the disinfectant in solution, no albuminous matter being present. In the actual practice of disinfection such conditions are never met with, the most important difference being the presence of albuminous material in greater or less quantity. Especially does this apply to the case of perchloride of mercury, which forms a definite combination with albumin, resulting in the formation of a comparatively inert albuminate of mercury, and thus, to maintain its efficiency, the strength of the solution would require to be increased in proportion to the amount of albuminous matter present.

Biniodide of mercury was brought into solution by the addition of iodide of potassium in quantity just sufficient to dissolve it, a stock solution of one part of biniodide in five hundred of distilled water being first prepared, from which the more dilute solutions were afterwards obtained by the addition of the requisite proportion of ordinary tap-water. The germicidal power of this drug is far superior to that of any other used in these experiments, and as it does not precipitate with albumin the results obtained should hold good even in the presence of albuminous material, thereby contrasting with the perchloride of mercury.

Chlorinated lime (commonly called "chloride of lime") is a white powder which is only partially soluble in water, and after standing for a little the insoluble portion falls to the bottom. This was kept in suspension in these experiments by frequent shaking of the glass vessel containing the threads immersed in the solution, but the germicidal power was not in any way altered by previously filtering the solution so as to remove this insoluble portion. In the stronger solutions of this substance not only are the bacilli on the threads all killed, but the threads themselves are destroyed, and in a short time almost all trace of them disappears.

Formaldehyde solutions were prepared from Schering's formalin, which is a forty per cent. solution of formaldehyde in water, the latter itself existing in the gaseous state.

Izal and medical izal, two coal products, are prepared by Messrs. Newton, Chambers & Co., the latter being a refined preparation of the former specially adapted to medical and surgical use. He found no difference between them as regards their germicidal properties.

Boric acid has no claim to be regarded as a disinfectant, since its saturated solution failed to destroy the bacillus coli communis in any of the allotted times.—*The Therapeutic Gazette.*

